# Comparative Evaluation of Bovine- and Porcine-Derived Xenografts in Rabbit Calvarial and Canine Mandibular Dehiscence Models

**DOI:** 10.3390/dj14040218

**Published:** 2026-04-08

**Authors:** Na Ri Seo, Hee Jeong Jang, Sung-Ho Lee, Bongju Kim, Dong-Wook Han

**Affiliations:** 1 Program in Neuroscience, Department of Dental Science, Graduate School, Seoul National University, Seoul 03080, Republic of Korea; tj0943@snu.ac.kr; 2 Institute of Nano-Bio Convergence, Pusan National University, Busan 46241, Republic of Korea; h78crom@naver.com; 3 Dental Life Science Research Institute, Seoul National University Dental Hospital, Seoul 03080, Republic of Korea; shlee79@snu.ac.kr; 4 Department of Cogno-Mechatronics Engineering, Pusan National University, Busan 46241, Republic of Korea; 5 School of Transdisciplinary Engineering, University College, Pusan National University, Busan 46241, Republic of Korea

**Keywords:** xenograft, bovine, porcine, guided bone regeneration, micro-computed tomography, dehiscence defect, calvarial defect

## Abstract

**Background:** This study compared two xenogeneic bone graft materials, A-Oss (bovine-derived) and The Graft (porcine-derived), using a rabbit calvarial defect model and a canine mandibular dehiscence-type defect model. **Methods:** Healing was evaluated at 6 and 12 weeks in rabbits and at 24 weeks in mongrel dogs. Micro-computed tomography quantified mineralized tissue fill (defect closure) in rabbits and, in dogs, the compartments classified as new bone and residual graft, together with vertical and horizontal volumetric maintenance. Hematoxylin and eosin (H&E) sections provided complementary qualitative observations. **Results:** In rabbits, defect closure did not differ between materials at 6 weeks (67.1 ± 12.7% vs. 70.2 ± 15.1%, *p* = 0.090) or 12 weeks (78.6 ± 5.9% vs. 72.3 ± 0.9%, *p* = 0.124). In dogs, new bone was similar between groups (43.5 ± 3.2% vs. 45.9 ± 1.1%, *p* = 0.208), whereas residual graft showed a numerical trend toward higher values with A-Oss (20.2 ± 3.5% vs. 13.3 ± 4.5%, *p* = 0.069). Vertical volume maintenance also trended higher with A-Oss (91.1 ± 1.6% vs. 87.8 ± 1.3%, *p* = 0.056), while horizontal maintenance was comparable (94.5 ± 1.8% vs. 91.4 ± 2.8%, *p* = 0.241). Histology in both models showed graft particles within the defect/augmented regions with surrounding eosinophilic matrix and intervening tissue spaces. **Conclusions:** Overall, both materials produced similar healing profiles across models, with small between-material differences most apparent in the canine dehiscence setting.

## 1. Introduction

Alveolar bone resorption following tooth extraction is a well-documented biological phenomenon that may affect the three-dimensional positioning of dental implants and the esthetic outcome of prosthetic rehabilitation. Experimental studies have reported pronounced changes in socket wall dimensions during early healing, with the buccal aspect often exhibiting greater resorption compared with the lingual plate [1]. In humans, systematic reviews have similarly described clinically relevant horizontal and vertical bone loss after extraction, suggesting that the magnitude and timing of dimensional change should be anticipated when planning implant placement [2,3]. These observations have supported the use of alveolar ridge preservation (ARP) and guided bone regeneration (GBR) procedures to attenuate post-extraction collapse and maintain ridge contour for prosthetically guided implant therapy.

Recent evidence syntheses have examined ARP outcomes across grafting materials and barrier approaches. A systematic review with meta-analyses reported that ARP with xenogeneic bone substitutes combined with resorbable socket-sealing materials reduced ridge resorption compared with spontaneous healing in the esthetic region, although the magnitude of benefit varied across studies [4]. A network meta-analysis likewise suggested that xenografts (XG) and allografts (AG) generally preserve horizontal and vertical ridge dimensions, whereas platelet concentrates may be favorable for histomorphometric outcomes such as new bone formation [5]. These reviews indicate that “dimension preservation” and “vital bone gain” do not always move in parallel, and that residual graft persistence can differ by material and protocol. On that basis, a head-to-head evaluation using standardized surgical conditions and quantitative 3D outcomes remains useful, particularly in dehiscence-type defects where contour maintenance is a primary clinical goal.

Xenogeneic grafts derived from bovine and porcine sources are widely used because of their osteoconductive properties, availability, and handling characteristics. Comparative clinical and preclinical evidence suggests that porcine-derived xenografts may perform similarly to bovine-derived xenografts in selected indications (e.g., sinus floor augmentation and alveolar ridge preservation), although observed differences are often small and context-dependent [6,7,8]. A systematic review and meta-analysis comparing porcine bone xenografts (PBX) and bovine bone xenografts (BBX) in maxillary sinus floor augmentation and ARP found no significant difference in newly formed bone, residual bone graft, or connective tissue percentages, indicating broadly comparable histomorphometric performance [7]. At the same time, interpretation of histomorphometric endpoints remains an active area of discussion. One review focusing on histomorphometric outcomes in ARP noted that both bovine- and porcine-derived xenografts were associated with clinically usable amounts of new bone, while residual graft percentages varied across studies and protocols [9]. This variability leaves room for differing clinical perspectives: residual xenograft particles are often interpreted as a slowly remodeling osteoconductive scaffold that can help maintain space and contour stability during healing, whereas more complete remodeling into vital bone may be prioritized when earlier turnover is desired.

Non-contained defects around implants or along the buccal aspect of the ridge can be challenging because graft stability and contour maintenance may be more technique-sensitive than in contained socket defects. Dehiscence-type peri-implant defects are clinically relevant because incomplete defect resolution after treatment has been associated with an increased risk of peri-implant disease during long-term follow-up [10]. Clinical studies of peri-implant dehiscence augmentation have evaluated stability and outcomes using different graft forms and membranes, including an L-shaped porcine-derived block bone substitute [11] and randomized comparisons of synthetic substitutes and bovine-derived materials [12]. In this context, direct head-to-head comparisons between clinically relevant xenograft products using standardized protocols and observation periods may help clarify whether material-specific behavior is detectable across different defect environments.

The present study compared two xenogeneic bone graft materials, A-Oss (bovine-derived) and The Graft (porcine-derived), using two independent in vivo models addressing complementary preclinical questions: a rabbit calvarial defect model to evaluate mineralized tissue fill over time (6 and 12 weeks) and a canine mandibular dehiscence-type defect model to assess new bone formation, residual graft persistence, and volumetric maintenance over an extended healing period (24 weeks). In rabbits, micro-CT quantified mineralized tissue fill within the calvarial defect ROI, whereas in the canine model, predefined buccolingual and mesiodistal sections were used to quantify compartment outcomes and volume maintenance; histology provided supportive qualitative assessment in both models. The study evaluated whether bovine- and porcine-derived xenografts differ in mineralized tissue fill, residual graft, and volumetric maintenance under standardized experimental conditions.

## 2. Materials and Methods

### 2.1. Study Design and Ethical Approval

Two independent in vivo experiments were conducted to compare two xenogeneic bone graft materials (A-Oss and The Graft) using (i) a rabbit calvarial defect model and (ii) a canine mandibular dehiscence defect model. Eight male New Zealand White rabbits (aged approximately 12–16 weeks, weighing 2.5–3.0 kg) and four male mongrel dogs (aged approximately 12–18 months, weighing 10–12 kg) were used in this study. Rabbits were purchased from DooYeol Biotech (Seoul, Republic of Korea), and dogs were purchased from Cronex (Hwaseong-si, Republic of Korea). Rabbit surgeries were performed at the Animal Research Center, Osstem Implant, in Seoul, Republic of Korea (Approval No. OST-IACUC2402). Canine surgeries were performed at the animal research facility of Cronex (Approval No. CRONEX-IACUC202101012). Animals were monitored throughout the experimental period for distress or abnormal behavior, including feeding patterns and activity levels.

### 2.2. Bone Graft Materials

Two xenogeneic bone graft materials were evaluated: A-Oss (Osstem Implant, Seoul, Republic of Korea) and The Graft (Purgo Biologics Inc., Seongnam-si, Republic of Korea). According to the manufacturers, the particle size range of A-Oss and The Graft was approximately 250–2000 μm and 250–2000 μm, respectively. The materials were applied to surgically created defects according to the protocols described for each model. In both models, outcome variables were assessed at the pre-specified healing periods, and analyses were conducted on harvested specimens.

### 2.3. In Vivo Models and Surgical Procedures

**Figure 1:**
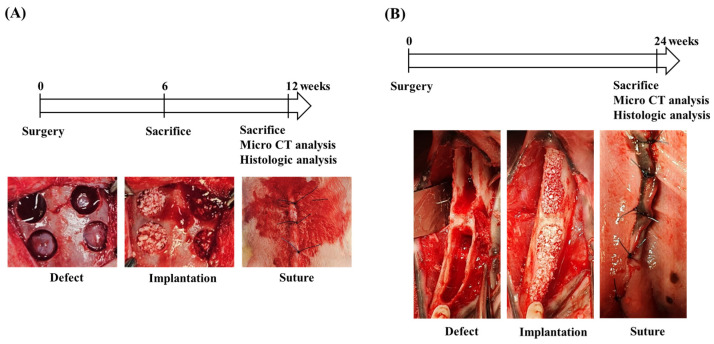
Experimental schedule and surgical procedures for two animal models. (**A**) Rabbit calvarial defect model: Surgery was performed at week 0, and animals were sacrificed at weeks 6 or 12 for micro-CT and histologic evaluation. (**B**) Mongrel dog mandibular dehiscence model: Surgery was performed at week 0, and animals were sacrificed at week 24. After sacrifice, micro-CT and histologic analysis were performed.

In the rabbit calvarial defect model, surgery was performed at week 0, and animals were sacrificed at weeks 6 or 12 for micro-CT and histologic analyses (Figure 1A). In the canine mandible dehiscence defect model, surgery was performed at week 0, and animals were sacrificed at week 24 for micro-CT and histologic analyses (Figure 1B).

#### 2.3.1. Rabbit Calvarial Defect Model

Four circular defects (6 mm in diameter) were created in the rabbit calvarium. Within each rabbit, two defects were randomly allocated to A-Oss and two to The Graft in a split design, enabling within-animal paired comparisons. The defects were filled with the assigned graft material, and specimens were collected after 6 or 12 weeks of healing for micro-CT imaging and H&E histology [13,14,15].

Prior to surgery, rabbits were anesthetized with an intramuscular injection of a mixture containing tiletamine and zolazepam (0.4 mL) and medetomidine (0.2 mL). The surgical site was shaved and disinfected using povidone–iodine solution and 70% ethanol. A 2 cm midline incision was made along the sagittal suture, and the periosteum was incised to expose the parietal bone. After bone grafting, the periosteum was closed using absorbable sutures (Maxon^®^; Covidien, Dublin, Ireland), and the skin was closed with non-absorbable sutures (Blue Nylon; AILEE Co., Busan, Republic of Korea). The surgical site was disinfected again using povidone–iodine solution. Postoperative analgesia and antibiotic prophylaxis were provided via intramuscular injection of meloxicam (0.15 mL) and Baytril^®^ (enrofloxacin, 0.6 mL; Bayer Vital, Leverkusen, Germany), respectively. Animals were sacrificed at 6 or 12 weeks postoperatively using CO_2_ inhalation, and harvested specimens were fixed in 4% paraformaldehyde for micro-CT and histologic analyses.

#### 2.3.2. Canine Mandibular Dehiscence-Type Defect Model

General anesthesia was induced with intramuscular tiletamine–zolazepam (Zoletil 50^®^; Virbac Korea, Seoul, Republic of Korea) and xylazine-HCl (Rompun^®^; Elanco Animal Health, IN, USA), followed by endotracheal intubation. Anesthesia was maintained with inhaled isoflurane (Ipharn^®^; Hana Pharm, Seoul, Republic of Korea). The surgical field was prepared and disinfected with chlorhexidine gluconate solution (Hexamedine^®^; Bukwang Pharmaceutical, Seoul, Republic of Korea).

Bilateral procedures were performed in each animal. The mandibular fourth premolar (PM4) and the first and second molars (M1 and M2) were extracted. Following flap elevation, standardized dehiscence-type defects were created by trimming the buccal aspect of the extraction sites/alveolar crest to a uniform height at the designated locations. Two defects were prepared per hemimandible (anterior and posterior), resulting in four defects per animal. Where applicable, the interradicular septum at the M1 site was preserved to maintain separation between the anterior and posterior defects. Defects were allocated to receive either A-Oss or The Graft so that each animal received both materials (two defects per material). Each defect was filled with the assigned graft material and covered with a resorbable collagen membrane (OssGuide; Hyundai Bioland Co., Cheongju-si, Republic of Korea), followed by suturing for closure.

Postoperative analgesia and antibiotic prophylaxis were administered intramuscularly with meloxicam (Metacam^®^; Boehringer Ingelheim Korea, Seoul, Republic of Korea) and enrofloxacin (Baytril^®^; Bayer Vital, Leverkusen, Germany) according to the facility protocol. Animals were monitored throughout the study period for general condition, feeding behavior, and activity. At 24 weeks, animals were euthanized under deep anesthesia followed by intravenous potassium chloride (KCl; Jeil Pharmaceuticals, Seoul, Republic of Korea). Mandibular segments containing the defect sites were harvested and fixed for subsequent micro-CT and histologic analyses.

### 2.4. Micro-CT Analysis

Defect closure, new bone formation, residual graft, and volume maintenance were quantified using micro-computed tomography (micro-CT; SMX-225CT, Shimadzu Co., Kyoto, Japan). Scanning was performed at 110 kV and 50 μA using a metal filter, and images were reconstructed at an isotropic voxel size of 8 μm. Reconstruction was performed with InspeXio software (Ver. 5.0.0.0) according to the manufacturer’s instructions.

For defect closure (rabbit calvarial model), the region of interest (ROI) was defined as the entire defect area delineated by the original defect margins on serial micro-CT slices. Mineralized tissue within the ROI was segmented using grayscale thresholding and expressed as a percentage of the total defect ROI [15].

For new bone formation and residual graft (canine mandibular model), buccolingual cross-sections were analyzed. The defect center was identified, and eight consecutive sections were selected at 0.1 mm intervals. In each section, the entire defect area was defined as the ROI. Newly formed bone and residual graft were separated by grayscale thresholding in ImageJ (version 1.54k, National Institutes of Health, Bethesda, MD, USA), and each compartment was calculated as a percentage of the ROI. Final values were obtained by averaging the eight section-level percentages.

**Figure 2:**
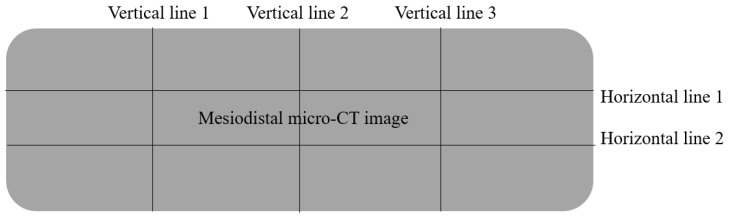
Schematic illustration of the micro-CT–based volume maintenance measurement in the canine mandibular dehiscence model. A representative mesiodistal micro-CT section is shown with the predefined reference grid used for quantitative assessment. Three vertical reference lines (vertical lines 1–3) were placed at one-quarter, one-half, and three-quarters of the defect width, and two horizontal reference lines (horizontal lines 1–2) were placed at one-third and two-thirds of the defect height. Measurements were performed on three sections (center and ±1 mm from the center). At each reference line, the graft-retained length was measured and expressed as a percentage of the corresponding original defect length. Horizontal volume maintenance was calculated as the mean of six measurements (two horizontal lines × three sections), and vertical volume maintenance as the mean of nine measurements (three vertical lines × three sections).

For volume maintenance (canine mandibular model), mesiodistal sections were analyzed using three representative cross-sections (center and ±1 mm from the center). In each section, the original defect length and the graft-retained length were measured along predefined reference lines. Two horizontal reference lines were positioned at one-third and two-thirds of the defect height, and three vertical reference lines were positioned at one-quarter, one-half, and three-quarters of the defect width (Figure 2). At each reference line, the graft-retained length was expressed as a percentage of the corresponding original defect length. Horizontal volume maintenance was calculated as the mean of six measurements (two horizontal lines × three sections), and vertical volume maintenance as the mean of nine measurements (three vertical lines × three sections). Mesiodistal sections were selected for volume maintenance analysis to enable standardized placement of reference grids and reproducible measurements across specimens. While buccolingual sections may offer more direct clinical interpretation of ridge width and height, the present approach prioritized consistency and comparability across multiple defects and animals.

### 2.5. Histological Processing and Evaluation

Fixed tissue samples were decalcified in 14% EDTA solution for 30 days (rabbit) or 4 months (canine). Following decalcification, specimens were dehydrated through a graded series of ethanol, cleared with xylene, embedded in paraffin, and sectioned at 4 μm thickness. Histological evaluation was performed using hematoxylin and eosin (H&E) staining [15].

### 2.6. Statistical Analysis

All data are presented as mean ± standard deviation. When multiple defects treated with the same material were present within an animal, values were averaged within each animal for each material, and the animal was treated as the experimental unit. Comparisons between A-Oss and The Graft were performed using a two-tailed paired Student’s *t*-test. Statistical analyses were conducted using Microsoft Excel, and *p* < 0.05 was considered statistically significant.

## 3. Results

### 3.1. Rabbit Calvarial Defect Closure

**Figure 3:**
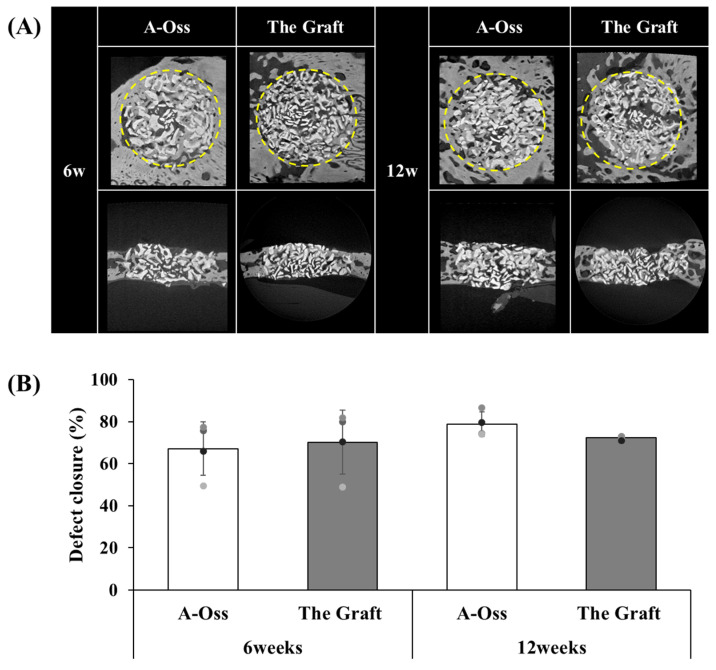
Micro-CT evaluation of defect closure in rabbit calvarial defects treated with A-Oss or The Graft. (**A**) Representative axial and sagittal micro-CT images at 6 and 12 weeks. The dotted outline indicates the original defect boundary used to define the region of interest. (**B**) Quantitative defect closure (%) at 6 and 12 weeks. Bars represent mean ± SD, and circles indicate individual animals (*n* = 4 per time point). *p*-values were obtained using a two-tailed paired Student’s *t*-test comparing A-Oss and The Graft at each time point.

Defect closure (%) was evaluated at 6 and 12 weeks in the rabbit calvarial defect model (Figure 3). At 6 weeks, defect closure was 67.09 ± 12.74% in the A-Oss group and 70.23 ± 15.10% in The Graft group (*p* = 0.090; Figure 3B). At 12 weeks, defect closure was 78.65 ± 5.85% in the A-Oss group and 72.33 ± 0.86% in The Graft group (*p* = 0.124; Figure 3B). No statistically significant differences were observed between materials at either time point.

### 3.2. Micro-CT Outcomes in the Canine Mandibular Dehiscence Defect Model

**Figure 4:**
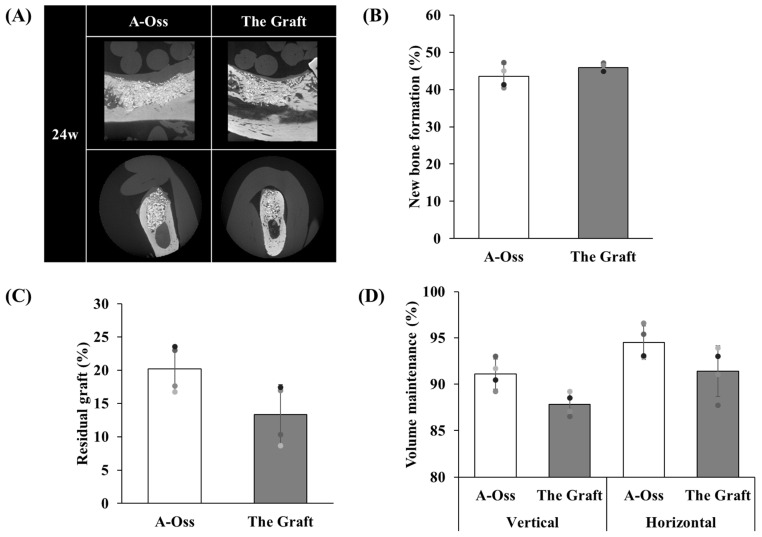
Micro-CT outcomes in the canine mandibular dehiscence-type defect model at 24 weeks. (**A**) Representative micro-CT images of augmented dehiscence-type defects prepared at extraction sites and treated with A-Oss or The Graft. Upper panels: mesiodistal sections used for volume maintenance measurements. Lower panels: buccolingual sections used for compartment quantification. (**B**) New bone formation (%) within the defect region of interest. (**C**) Residual graft (%) within the same region of interest. (**D**) Vertical and horizontal volume maintenance (%) assessed on mesiodistal sections using predefined reference lines. Bars represent mean ± SD; individual animals are shown as overlaid data points. *p*-values were obtained using two-tailed paired Student’s *t*-tests comparing A-Oss and The Graft for each outcome.

Micro-CT outcomes at 24 weeks are presented (Figure 4). Representative buccolingual sections and mesiodistal sections are shown (Figure 4A), and quantitative outcomes are summarized (Figure 4B–D). New bone formation was 43.50 ± 3.22% in the A-Oss group and 45.92 ± 1.12% in The Graft group (*p* = 0.208; Figure 4B). Residual graft was 20.21 ± 3.54% in A-Oss and 13.34 ± 4.49% in The Graft (*p* = 0.069; Figure 4C). Vertical volume maintenance was 91.09 ± 1.62% in A-Oss and 87.83 ± 1.25% in The Graft (*p* = 0.056; Figure 4D), and horizontal volume maintenance was 94.48 ± 1.81% in A-Oss and 91.41 ± 2.75% in The Graft (*p* = 0.241; Figure 4D).

### 3.3. Histologic Observations in the Rabbit Calvarial Defect Model

**Figure 5:**
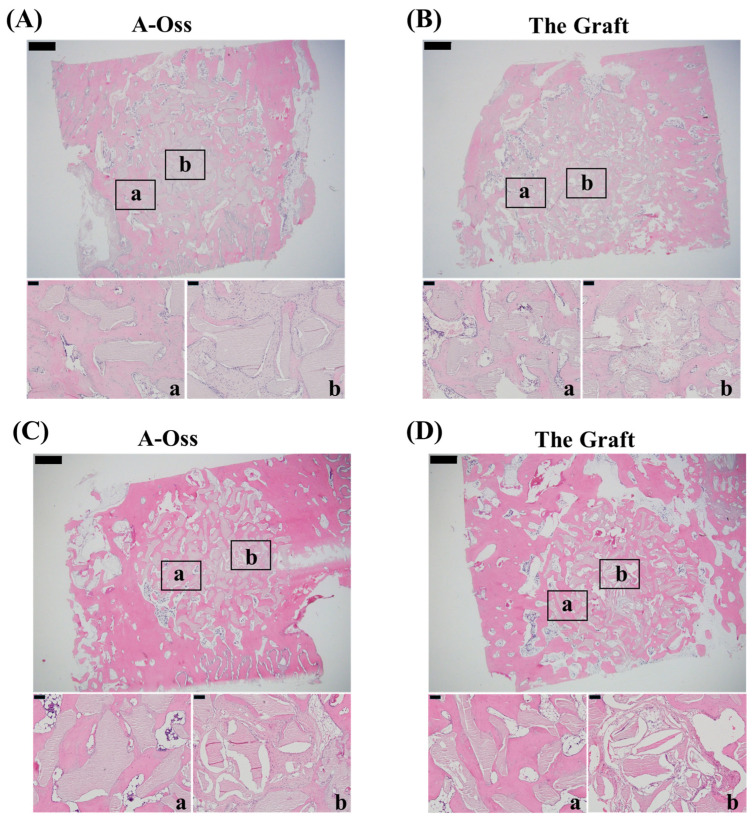
Representative H&E-stained sections of rabbit calvarial defects grafted with A-Oss or The Graft: (**A**) 6 weeks, A-Oss; (**B**) 6 weeks, The Graft; (**C**) 12 weeks, A-Oss; (**D**) 12 weeks, The Graft. Upper panels show low-magnification views of the defect region (scale bar = 1 mm). Boxed areas (a and b) in each low-magnification image correspond to the higher-magnification views shown below (scale bar = 0.1 mm). In all panels, graft particles are present within the defect area, with an adjacent eosinophilic matrix and intervening tissue spaces.

Representative H&E-stained sections of the rabbit calvarial defects at 6 and 12 weeks are shown in Figure 5. At 6 weeks (Figure 5A,B), graft particles were present within the defect region, with intervening tissue spaces containing eosinophilic matrix and scattered basophilic cellular elements. At 12 weeks (Figure 5C,D), the defect area similarly contained graft particles and surrounding eosinophilic tissue. In the presented sections, the intervening eosinophilic tissue between particles appeared, in some fields, to span more continuously across the defect region compared with the 6-week specimens, although discrete interparticle spaces remained evident. Higher-magnification views from the boxed regions (a and b) illustrate the local tissue appearance adjacent to graft particles at each time point.

### 3.4. Histologic Observations in the Canine Mandibular Dehiscence Defect Model

**Figure 6:**
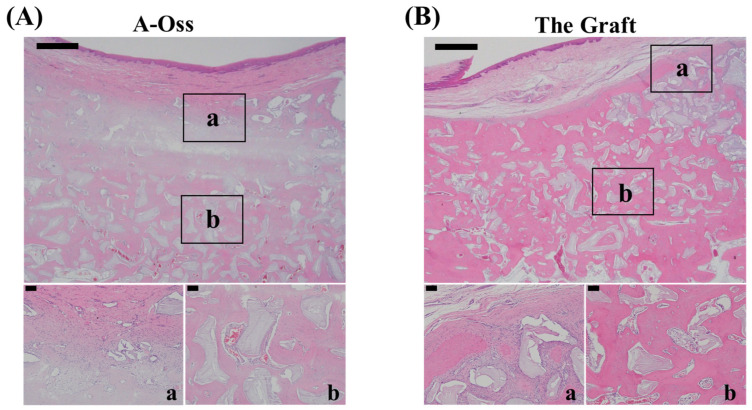
Representative H&E-stained sections of canine mandibular dehiscence defects at 24 weeks grafted with A-Oss or The Graft. (**A**) A-Oss. The upper panel shows a low-magnification view of the augmented region (scale bar = 1 mm), and boxed areas a and b correspond to the higher-magnification views shown below (scale bar = 0.1 mm). Graft particles are present within the augmented area, with adjacent eosinophilic matrix and intervening tissue spaces. (**B**) The Graft. The upper panel shows a low-magnification view of the augmented region (scale bar = 1 mm), and boxed areas a and b correspond to the higher-magnification views shown below (scale bar = 0.1 mm). Graft particles are present within the augmented area, with an adjacent eosinophilic matrix and intervening tissue spaces.

Representative H&E staining sections obtained at 24 weeks are shown in Figure 6. In both the A-Oss and The Graft groups, graft particles were present within the augmented region, surrounded by an eosinophilic matrix with intervening tissue spaces. In the presented sections, the superficial portion of the augmented area was covered by a fibrous-appearing tissue layer, and graft particles were distributed beneath this layer throughout the defect region. Higher-magnification views from the boxed regions (a and b) illustrate the local tissue appearance adjacent to graft particles within the superficial and central portions of the augmented area.

## 4. Discussion

### 4.1. Overview and Comparative Framework

This study compared two xenogeneic bone substitutes (A-Oss and The Graft) using a rabbit calvarial defect model with 6 mm circular defects evaluated at 6 and 12 weeks, and a canine mandibular dehiscence-type defect model assessed at 24 weeks. Both materials function primarily as osteoconductive scaffolds, and recent clinical trials comparing porcine- and bovine-derived xenografts in augmentation procedures have reported broadly comparable histomorphometric and radiographic outcomes, though endpoint definitions and defect characteristics can influence the magnitude of observed differences [6,7,8].

### 4.2. Defect Closure in the Rabbit Calvarial Model

In the rabbit calvarial model, the proportion of mineralized tissue within the defect region of interest increased from 6 to 12 weeks in both groups, indicating continued mineralized tissue fill over the observation window. This time-dependent increase is consistent with ongoing mineralized tissue ingrowth during early healing rather than an early plateau. The 6 mm rabbit calvarial defect model is widely used in preclinical bone regeneration studies and is often considered a critical-size defect; however, its classification may vary depending on the observation period, healing duration, and experimental conditions [13,14,15]. Partial spontaneous healing may occur over extended periods, and therefore, this model should be interpreted as a standardized preclinical defect model rather than as an absolutely non-healing defect. In this contained defect environment, the between-material differences at each time point were small and did not reach statistical significance, suggesting that early-to-intermediate mineralized tissue fill was comparable for the two xenografts under the present protocol.

### 4.3. New Bone Formation and Graft Remodeling in the Canine Dehiscence Model

In the canine mandibular dehiscence model at 24 weeks, micro-CT–derived outcomes showed similar proportions of the compartment classified as new bone between materials, while residual graft was numerically higher in the A-Oss group. Vertical volume maintenance showed a similar borderline trend (91.1% vs. 87.8%), whereas horizontal maintenance was comparable. Although these differences were not statistically significant with the current sample size, the direction of the trends is clinically relevant because particulate xenografts are often selected for space maintenance in non-contained defects. One plausible explanation is that manufacturing-related parameters (e.g., mineral phase and surface characteristics) can affect in vivo resorption kinetics and persistence of graft particles [16]. Particle size may influence graft resorption kinetics, surface area availability, and cellular infiltration. Smaller particles generally provide increased surface area but may resorb more rapidly, whereas larger particles may contribute to prolonged space maintenance. Such differences could partially explain the observed trends in residual graft and volumetric stability. Because the nominal particle size ranges reported by the manufacturers were similar for both materials, the observed differences in residual graft and volumetric maintenance are unlikely to be explained by particle size alone and may also reflect other material-related properties. Also, dehiscence outcomes can be sensitive to wound stability and membrane sealing, which influence volumetric stability alongside material behavior [17].

### 4.4. Clinical Context and Translational Considerations

Two contextual points frame the present findings. First, systematic reviews generally document substantial ridge shrinkage following tooth extraction without intervention, supporting the rationale for ridge preservation when contour maintenance is needed for implant positioning [3]. Second, evidence syntheses suggest that xenografts tend to attenuate ridge dimensional loss compared with unassisted healing, though the material that optimizes ridge dimensions is not necessarily the material that maximizes newly formed bone histologically [4,7]. This distinction may be clinically relevant because residual graft persistence can be interpreted as reflecting slower remodeling kinetics, whereas the vital bone fraction represents a separate biological endpoint; these domains may not change in parallel across materials or settings [7,18].

The rabbit calvarial model represents a non-load-bearing environment, whereas the canine mandibular model reflects a load-bearing condition. Mechanical loading may influence graft stability, remodeling dynamics, and volumetric maintenance. Therefore, differences between models should be interpreted in the context of biomechanical environments.

The present two-model dataset provides controlled preclinical information under standardized surgical and analytical conditions. However, direct clinical extrapolation requires caution given differences between preclinical models and human clinical scenarios [6,7,8].

### 4.5. Analytical Considerations for Micro-CT Quantification

Micro-CT enables nondestructive, three-dimensional quantification; however, separating the compartment classified as new bone from residual graft can depend on the segmentation approach and threshold selection. In particulate xenograft studies, grayscale overlap between mineralized particles and newly mineralized tissue may introduce uncertainty in compartment assignment, particularly when between-group differences are small [19]. Methodological studies comparing micro-CT–derived outcomes with histologic or histomorphometric assessments have reported that the level of agreement can vary with the definitions applied and the sampling strategy used [20]. For these reasons, it can be helpful to report segmentation criteria explicitly and, where feasible, to interpret compartment-level micro-CT outcomes alongside histologic observations, especially when such outcomes are central to the study’s inferences [19,20].

### 4.6. Study Limitations

Several limitations should be acknowledged. First, the rabbit and canine experiments were conducted as separate studies with different anatomical sites, defect configurations (contained vs. non-contained), and healing periods; the two models therefore address complementary questions rather than providing a single directly comparable dataset. Second, the canine analysis was performed at the animal level by averaging multiple defects per dog, and the small number of animals limits power for detecting modest differences and for evaluating multiple micro-CT endpoints. Third, compartment assignment on micro-CT (new bone vs. residual graft) is inherently influenced by thresholding when radiodensities overlap, and histology in the present work was qualitative rather than ROI-matched histomorphometry. Larger cohorts and/or mixed-effects analyses that incorporate the within-animal structure, together with histomorphometric measurements aligned to the micro-CT regions of interest, would strengthen inference and improve compartment validation [19,20].

From a clinical perspective, buccolingual sections may provide more intuitive information regarding ridge width and height. However, mesiodistal sections were used in this study to ensure standardized and reproducible measurements using predefined reference grids. This methodological choice may limit immediate clinical interpretability and should be considered when extrapolating the findings.

Healing capacity differs between species, with rabbits generally exhibiting faster bone turnover and regeneration compared with larger animals such as dogs. Therefore, the faster mineralized tissue fill observed in the rabbit model should not be directly extrapolated to clinical scenarios without caution.

From a translational perspective, extended observation windows and incorporation of planning-relevant endpoints may help clarify whether differences in graft persistence or contour metrics are associated with differences in contour availability or site development measures relevant to implant therapy [7,21].

## 5. Conclusions

Across two preclinical models, A-Oss and The Graft produced comparable defect closure in rabbit calvarial defects and similar new bone formation in canine mandibular dehiscence defects at the evaluated time points. Residual graft percentage and vertical volume maintenance showed modest numerical differences favoring A-Oss, but these did not reach statistical significance in two-tailed paired Student’s *t*-tests.

Histologic observations in both models demonstrated graft particles within the defect/augmented regions accompanied by surrounding tissue matrix and intervening spaces, supporting a conservative interpretation that both materials integrated within the healing environment. Future studies with larger cohorts and coordinated histomorphometric analyses aligned to micro-CT regions of interest may help determine whether modest differences in graft persistence are reproducible and whether they are associated with differences in volumetric maintenance in dehiscence-type defects.

## Data Availability

The datasets generated during and analyzed during the current study are available from the corresponding authors on reasonable request.
